# Efficacy and feasibility of virtual reality-based cognitive tele-rehabilitation in Parkinson's disease: A pilot randomized controlled trial on patients and caregivers

**DOI:** 10.1177/20552076251376534

**Published:** 2025-09-25

**Authors:** Maria Grazia Maggio, Amelia Rizzo, Alessandra Benenati, Fabio Giambò Mauro, Antonino Cannavò, Paolo De Pasquale, Rosaria De Luca, Angelo Quartarone, Rocco Salvatore Calabrò

**Affiliations:** 1 120349 IRCCS Centro Neurolesi Bonino Pulejo, Messina, Italy; 2 Department of Clinical and Experimental Medicine, 18980 University of Messina, Messina, Italy; 3 Department of Psychology “Renzo Canestrari”, 9296 University of Bologna, Bologna, Italy; 4 213334 A.O.U. Policlinico “G. Martino”, Messina, Italy

**Keywords:** Caregiver strain, cognitive functions, neurorehabilitation, telemedicine, virtual reality

## Abstract

**Objective:**

Parkinson's disease (PD) is a progressive neurodegenerative disorder that not only impairs motor functions but also may lead to significant cognitive decline, greatly affecting the quality of life for both patients and their caregivers. This study aims to evaluate the feasibility and potential effectiveness of cognitive tele-rehabilitation (TR) plus virtual reality (VR), compared to traditional home-based rehabilitation methods, focusing on cognitive outcomes, quality of life, usability, and the impact on caregiver strain in patients with PD.

**Methods:**

In this pilot randomized controlled trial, 20 PD patients and their primary caregivers were randomly assigned to either a control group (CG) receiving traditional cognitive training or an experimental group (EG) undergoing cognitive TR using a VR rehabilitation system, with assessments conducted before and after the 8-week intervention.

**Results:**

TR was rated as highly usable (System Usability Scale mean score: 84.3 ± 9.5). Post-intervention analyses showed significant improvements in global cognitive functioning (Montreal Cognitive Assessment (MoCA): *p* = 0.002) and quality of life (Parkinson's Disease Questionnaire (PDQ)-8: *p* = 0.002) in the EG. The CG also showed improvements in MoCA (*p* = 0.008) and PDQ-8 (*p* = 0.016), although to a lesser extent than the EG. A reduction in caregiver burden was observed in both groups but did not reach statistical significance. Analysis of *Δ* scores revealed a significantly greater MoCA improvement in the EG compared to the CG (*p* = 0.019). Statistically significant differences were found between the groups in the Family Strain Questionnaire and PDQ-8 *Δ* scores.

**Conclusion:**

These findings suggest that cognitive TR, particularly when integrated with VR, might be useful in improving cognitive function and quality of life in PD patients and could play a key role in future PD management strategies, potentially easing the burden on caregivers.

## Introduction

Parkinson's disease (PD) is one of the most common neurodegenerative disorders, affecting approximately 10 million people worldwide and carrying an economic cost estimated in billions of dollars annually to the healthcare system.^1,2^ PD is characterized by the progressive degeneration of dopaminergic neurons, primarily manifesting as motor symptoms such as tremors, rigidity, and bradykinesia.^1^ However, the disease may also lead to cognitive decline, which can progress to dementia, with significant concerns for patients and their caregivers.^2–4^ This cognitive deterioration directly affects the patient's autonomy and overall quality of life, placing a considerable burden on caregivers who face increasing challenges in managing the disease in daily living.^2–5^ The experiences of caregivers are critical to understanding the broader impact of PD.^5–7^ Caregivers often face emotional, psychological, and physical challenges, as they must balance the demands of care with their personal lives.^6^ The stress and exhaustion associated with caregiving can lead to burnout, making the availability of support systems, such as caregiver support groups and respite care services, essential for maintaining their well-being. Some studies highlight the profound effects that caregiving can have on mental health, reinforcing the need for comprehensive support mechanisms.^7–10^ Nonetheless, while caregiver support is essential, it is equally important to address the accessibility of cognitive rehabilitation services for patients themselves. Individuals with PD often face multiple barriers to accessing in-person interventions, including reduced mobility, fatigue, transportation difficulties, and geographical distance from specialized centers. These obstacles limit consistent participation in conventional cognitive rehabilitation programs.^7^ In this context, cognitive tele-rehabilitation (TR) has emerged as an innovative strategy to overcome such barriers and enhance the continuity of care.^11^ Digital platforms can deliver cognitive exercises and remotely monitor patient progress, overcoming geographical and logistical barriers, and restricting access to traditional rehabilitation services.^11–17^ Among these technologies, virtual reality (VR) could integrate into TR, providing immersive and interactive environments that enhance cognitive engagement and therapeutic outcomes.^18^ VR has shown significant promise in simulating real-life scenarios where patients can practice cognitive tasks in a safe and controlled setting, leading to greater improvements in cognitive function compared to traditional methods.^12,13^ The combination of TR and VR offers unique advantages, such as the ability to remotely deliver personalized therapy that adapts in real-time to the patient's performance. This is achieved through the virtual reality rehabilitation system’s (VRRS) automated feedback mechanisms and the therapist's ability to remotely modify exercises via the central Cockpit interface, making the treatment dynamic and responsive even in home settings, without the need for in-person supervision.^11,12^ This synergy between TR and VR is proving to be one of the most promising developments in the management of cognitive symptoms in PD. Recent studies suggested that cognitive TR, especially when combined with VR, may be effective in maintaining or improving cognitive abilities in PD patients, while also providing continuous support that can help alleviate the burden on caregivers.^14–16^ This support comes from a reduction in the need to travel to rehabilitation centers, increased patient autonomy, and the ability for caregivers to rely on a structured, remotely supervised program, which reduces their direct involvement in daily rehabilitation activities.^12,14^ Unlike traditional home-based rehabilitation methods, TR allows for more frequent monitoring and timely adjustments to therapies, contributing to more personalized and flexible management of the patient.^12,15^

This pilot study evaluated the feasibility, usability, and potential efficacy of cognitive TR plus semi-immersive VR, compared to traditional home-based rehabilitation. The aim was to assess effects on cognition, quality of life, usability and caregiver burden in patients with PD.

## Materials and methods

### Study population and randomization

This study included 20 PD patients attending the Robotic and Behavioral Neurorehabilitation Unit of the IRCCS Centro Neurolesi “Bonino-Pulejo” in Messina, Italy, between October 2019 and March 2020, and their primary caregiver.

All patients were randomly assigned, utilizing block randomization with a block size of 2 × 2, to two groups: the CG (control group), consisting of 10 patients who received traditional cognitive training, or the EG (experimental group), consisting of 10 patients who underwent TR training with VRRS. Randomization was performed by an independent researcher not involved in assessments or interventions, using a computer-generated sequence created with free online software Sealed Envelope (https://www.sealedenvelope.com). Block randomization was chosen due to the small sample size, in order to minimize group size imbalance and ensure equal allocation throughout the recruitment process.

Inclusion criteria were (i) PD patients diagnosed according to the Movement Disorders Society (MDS) diagnostic criteria^19^; (ii) aged between 40 and 80 years; (iii) at least 5 years of formal education; (iv) Hoehn and Yahr disease stage <3 (VRRS device needs to be used via a touchscreen, which requires sufficient motor skills); and (v) presence of a primary caregiver who has consented to participate in the study. Although a minimum number of caregiving hours was not set as an inclusion criterion, all caregivers were actively involved in the patient's daily care. They were typically cohabiting spouses or adult children, providing continuous support compatible with the patient's Hoehn and Yahr stage (≤3).

The exclusion criteria were: (i) presence of psychiatric disorders (major depression, psychosis, anxiety disorders); (ii) dementia, diagnosed according to the MDS diagnostic criteria^20^; (iii) severe motor or sensory impairments that could interfere with the use of the VRRS; and (iv) current participation in another cognitive or physical rehabilitation program.

Given the exploratory nature of this pilot study, no formal power analysis was performed. The sample size was determined based on feasibility constraints and aligned with established methodological recommendations for pilot trials, which commonly suggest enrolling approximately 10 participants per group. This approach allows for the assessment of feasibility parameters and preliminary effect size estimates to inform future larger-scale randomized controlled trials.^21^

The study was conducted in accordance with the 1964 Helsinki Declaration and approved by the Local Ethics Committee of IRCCS Centro Neurolesi “Bonino-Pulejo,” Messina, Italy (ID: 08/2018). Each study participant provided informed consent. This trial is part of a multicenter research project registered on ClinicalTrials.gov (Identifier: NCT05842577), titled “Efficacy of Non-immersive Virtual Reality-based Telerehabilitation in Parkinson's Disease: A Multicentre Randomized Controlled Trial.” The trial aims to evaluate the effectiveness of home-based cognitive telerehabilitation using virtual reality in individuals with Parkinson's disease. The present article specifically reports the results from the cognitive rehabilitation arm conducted at the IRCCS Centro Neurolesi “Bonino-Pulejo,” focusing on feasibility, usability, and cognitive and quality of life outcomes.

### Procedures

Each patient underwent an assessment before (T0) and immediately after (T1) the rehabilitation treatment, using the Montreal Cognitive Assessment (MoCA),^21^ the Family Strain Questionnaire (FSQ),^22^ the Parkinson's Disease Questionnaire (PDQ-8) total score,^23,24^ and the System Usability Scale (SUS).^25^ Outcome assessments were conducted by blinded evaluators who were not involved in the intervention delivery or group allocation.

The MoCA is a widely used tool designed to evaluate cognitive function across multiple domains, including attention, memory, language, and executive functions.^21^ Given the exploratory nature of this pilot study and in order to reduce patient burden, a more extensive neuropsychological battery was not administered. We adopted the Italian validated version of the MoCA, using the normative data and scoring procedures proposed by Santangelo et al.^26^

The FSQ assesses the level of strain experienced by the caregiver, focusing on the emotional and practical challenges of caregiving. The PDQ-8 is a shorter form of the PDQ-39, designed to measure the quality of life in individuals with PD, evaluating areas such as mobility, emotional well-being, and social support.^24^ The SUS is a 10-item scale designed to assess the usability of a device. Each item is rated on a Likert scale from 1 (strongly disagree) to 5 (strongly agree), with positive and negative statements to minimize bias. The final score, which ranges from 0 to 100, indicates overall usability: scores above 80.3 indicate excellent usability, whereas scores between 68 and 80.3 indicate good usability with room for improvement. Finally, scores below 68 indicate significant usability problems.^25^

In addition, the occurrence of any adverse events was monitored throughout the intervention period to evaluate the safety and feasibility of the treatment.

All study participants received cognitive rehabilitation three times a week for 8 weeks for a total of 24 sessions, with each session lasting approximately 45 min. The sessions were designed to target-specific cognitive domains, with the complexity of exercises progressively increasing based on individual performance. Progress was established by correct responses (i.e. achieving 9 out of 10 correct answers) and minimizing errors (less than one error per session). Both groups participated in the same number of neurorehabilitation sessions.

In Table 1, we summarized the key characteristics of the cognitive rehabilitation activities delivered to each group. Both interventions targeted similar cognitive domains, including attention, memory, and executive functions, but differed in the modality of delivery.

**Table 1.** Summary of the cognitive rehabilitation activities administered in the control group and experimental group.

Cognitive domain	Experimental group	Control group
Attention	Visual and auditory attention tasks in VR environment	Cancellation tasks, trail making paper versions
Memory	Virtual navigation with object recall; memory games with real-time feedback	Word list recall, story recall, paired-associate learning
Executive functions	Problem-solving and planning tasks with adaptive difficulty	Mazes, category fluency, planning paper tasks
Language	Naming, sentence completion and comprehension in interactive VR scenarios	Naming tasks, verbal fluency, reading comprehension
Visuospatial skills	3D figure rotation and object manipulation in VR	Figure copying, visual discrimination
Frequency and duration	3 sessions/week, 45 min each, for 4 weeks (remote supervision via Cockpit)	3 sessions/week, 45 min each, for 4 weeks (self-guided)

VR: virtual reality.

The CG received conventional cognitive rehabilitation based on validated paper-and-pencil exercises targeting the same cognitive domains as the EG (e.g. memory, attention, executive functioning). Exercises were standardized and self-administered under caregiver supervision, without real-time feedback or adaptive modifications. The exercises were completed twice a week for 6 weeks, following a predefined protocol. A trained therapist provided weekly remote contact to monitor adherence, clarify doubts, and support the progression of activities. Details of the rehabilitation content for both groups are summarized in Table 1.

**Figure 1.**
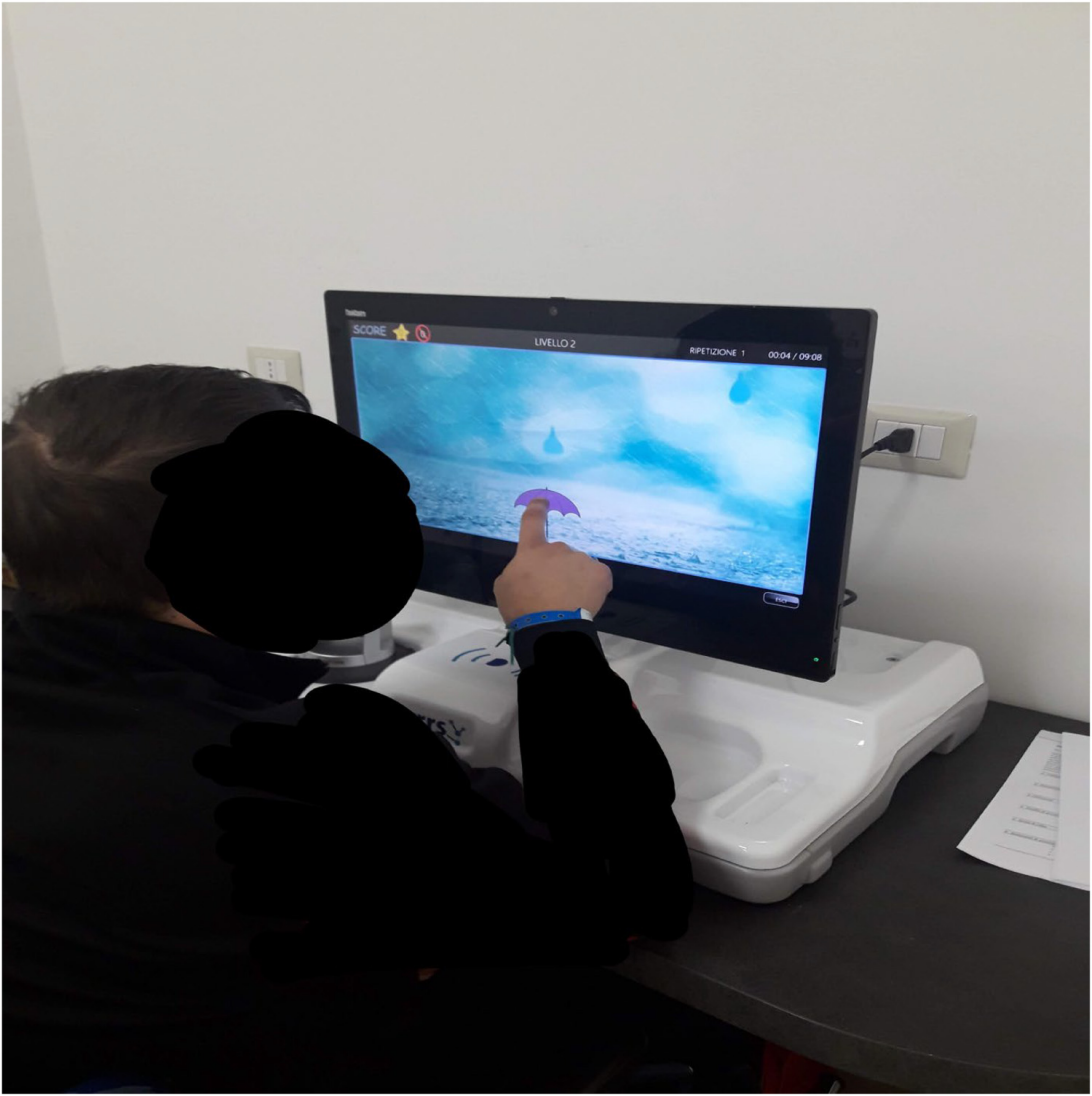
PD patient performing a cognitive exercise at home using the VRRS device. PD: Parkinson's disease; VRRS: virtual reality rehabilitation system.

The EG underwent cognitive TR using the semi-immersive VRRS, which provided real-time task adaptation and remote therapist supervision. VRRS (Khymeia, Padua, Italy) is an internationally patented, Class I-certified medical device.^27^ This system is known for its user-friendliness, high customization capabilities, automatic reporting, and TR functions. The VRRS uses a semi-immersive VR environment and provides a wide range of over 50 cognitive exercises designed to target-specific functions such as memory, attention, language, and executive functions. These exercises are primarily conducted on a VR screen, where patients interact with two-dimensional scenarios using the touchscreen for precise control. In this study, a home-based TR system was employed, where patients performed cognitive exercises on a VRRS screen under the remote supervision of a psychological therapist (Figure 1).

**Figure 2.**
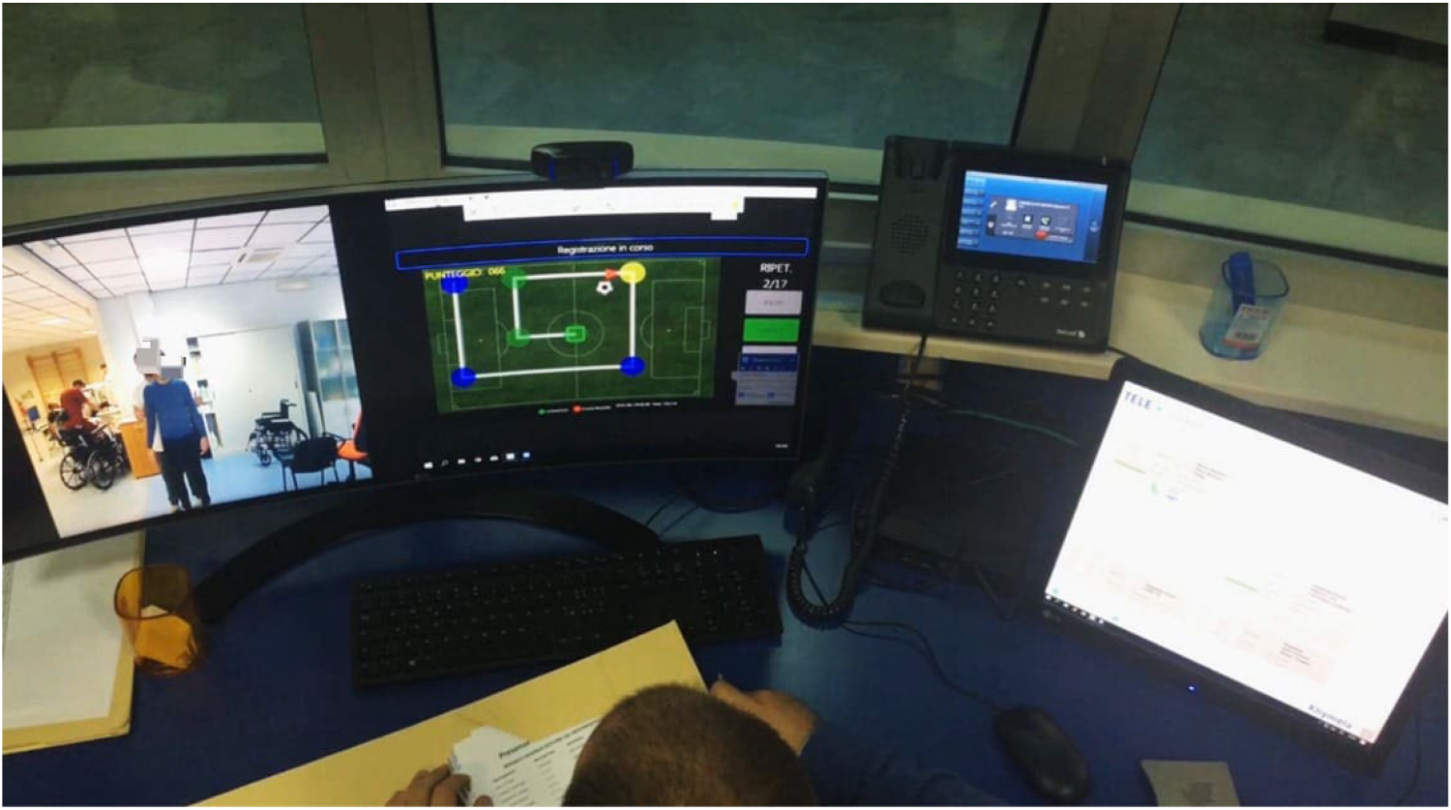
Cockpit station in our institute from which the therapist monitors the patient.

The therapist and patient were connected in real time from different locations, allowing for personalized guidance and continuous monitoring, all from the comfort of the patient's home. The therapist delivered the training through a Cockpit, a centralized interface within the VRRS. This Cockpit allows the therapist to customize and adjust the cognitive exercises remotely, track the patient's performance in real time, and make immediate modifications based on the patient's progress. Through the Cockpit, the therapist can also communicate directly with the patient, providing instant feedback and support, ensuring the exercises are tailored to the patient's evolving needs and capabilities (Figure 2).

### Statistical analysis

The data were analyzed using the latest version of Jamovi 2.4.14 (Jamovi Project, Sydney, Australia), with statistical significance set at *p* < 0.05. Descriptive statistics were presented as mean ± standard deviation, and categorical variables were reported as frequencies and percentages. Given the small sample size, non-normality of distribution was assumed, and non-parametric tests were used. Differences between the two groups (EG and CG) were assessed using the Mann–Whitney *U* test for patient's characteristics, such as age, education level, disease duration, and baseline of the tests. For gender distribution, Fisher's exact test was applied due to the categorical nature of the variable and the presence of low expected frequencies. Comparisons of clinical test results between two time points (T0 and T1) were conducted using the Wilcoxon signed-rank test. In addition, delta scores (*Δ* = T1 − T0) were calculated for each participant, and between-group differences in these delta scores were analyzed using the Mann–Whitney *U* test. Finally, a sensitivity analysis was conducted using G*Power (3.1.9.7) to estimate the minimum detectable effect size, given the study's sample size (*n* = 10 per group), an alpha level of 0.05, and a power of 0.80. The analysis, based on a two-tailed *t*-test for independent means and assuming a normal distribution, indicated that the minimum detectable effect size was Cohen's *d* = 1.36.

## Results

All patients completed the treatment without experiencing any adverse effects, and there were no dropouts throughout the study. For detailed demographic and clinical characteristics of the sample, refer to Table 2.

**Table 2.** Demographic characteristic of the sample.

	EG	CG	All	*p*-value	Type
Subjects	10	10	20	-	
Age (years)	63.6 ± 2.6	63.8 ± 2.0	63.7 ± 2.3	0.65	U
Male	6 (60%)	6 (60%)	12 (60%)	1.00	Fisher
Education (years)	13.1 ± 3.9	12.3 ± 3.4	12.7 ± 3.6	0.60	U
Disease duration	5.80 ± 1.3	5.60 ± 1.6	5.7 ± 1.4	0.73	U
Hoehn and Yahr stage	2.3 ± 0.3	2.2 ± 0.2	2.2 ± 0.3	0.398	U

EG: experimental group; CG: control group.

Mean ± standard deviation was used to describe continuous variables; proportions (numbers and percentages) were used to describe categorical variables. Fisher (Fisher's exact test); U (Mann–Whitney *U* test).

Moreover, a total of 20 primary caregivers (one per patient) participated in the study. The caregiver sample included 12 females (60%) and 8 males (40%), with a mean age of 60.1 ± 10.1 years and a mean education level of 12.2 ± 3.1 years.

The patients were matched by gender between the two groups (*p* value = 1.00). However, no significant statistical differences were observed between the groups at baseline regarding age (*p* value = 0.65), education (*p* value = 0.60), and disease duration (*p* value = 0.73). Regarding neuropsychological tests, no significant differences were observed at baseline in MoCA (*p* value = 0.78), PDQ-8 (*p* value = 0.57), and FSQ (*p* value = 0.08).

Post-intervention analysis showed significant within-group improvements (Table 3). In the EG, significant improvements were observed in both global cognitive functioning (MoCA, *p* = 0.002) and quality of life (PDQ-8, *p* = 0.002). In the CG, significant improvements were also found in global cognitive functioning (MoCA, *p* = 0.008), and in quality of life (PDQ-8, *p* = 0.016); however, the improvement was still lower compared to the EG. Finally, a likely reduction in caregiver burden was observed in both groups, although the change did not reach statistical significance.

**Table 3.** Statistical comparisons of neuropsychological evaluations.

Clinical assessment	EG		*p*-value	CG		*p*-value	EG	CG	*p*-value
	T0	T1		T0	T1		*Δ* = T1 − T0	*Δ* = T1 − T0	
MoCA	24 (23–25)	26.5 (25–27)	**0**.**002**	23.5 (23–24)	25 (25–25)	**0**.**008**	2 (2–3)	1 (1–2)	**0**.**019**
FSQ	11.5 (10–12.7)	11 (8.5–12)	0.352	9 (8–10)	8 (7–10.5)	0.086	−0.5 (−2 to 1)	−1 (−2 to 0)	0.818
PDQ-8	12.5 (10.5–14)	7.5 (5.5–9)	**0**.**002**	11 (10.2–12.7)	8.5 (5.2–9.7)	**0**.**016**	−5 (−7 to −4)	−5 (−5 to −3)	0.481

CG: control group; EG: experimental group; FSQ: Family Strain Questionnaire; MoCA: Montreal Cognitive Assessment; PDQ-8: Parkinson's Disease Questionnaire.

Scores are in median (first-third quartile); significant differences are in bold.

Between-group differences (Table 3) were assessed through *Δ* score analysis, which revealed a statistically significant difference in MoCA performance (*p* = 0.019), with greater improvement observed in the EG. No statistically significant differences were found between groups in the FSQ and PDQ-8 *Δ* scores.

Moreover, it is worth noting that, given the limited sample size, the study was powered to detect only large effects (Cohen's *d* ≥ 1.36), as indicated by the sensitivity analysis. Therefore, smaller yet potentially meaningful effects may have gone undetected.

Finally, the SUS scores indicated that the training was well accepted by the users, with a mean score of 84.3 ± 9.5.

Regarding clinical significance, the mean change in MoCA scores in the EG exceeded the commonly accepted minimal clinically important difference (MCID) threshold of 2 points, suggesting that the observed improvement was not only statistically significant but also clinically meaningful.^28,29^ For PDQ-8, the mean change approached, but did not exceed, the proposed MCID threshold (approximately 5–8 points), indicating a possible trend toward clinical relevance.^5^

## Discussion

Our study evaluated the feasibility and impact of TR compared to traditional home-based cognitive rehabilitation in patients with PD. TR proved to be highly feasible, as reflected by high SUS scores. Notably, participants in the EG showed greater improvements in global cognitive functioning and quality of life than those in the CG, as measured by MoCA and PDQ-8 scores. While CG also demonstrated significant gains in cognitive functioning, improvements in quality of life did not reach statistical significance.

These results suggest that, although both methods were beneficial, the EG showed more pronounced improvements in cognitive function, particularly in MoCA scores, as indicated by within-group analyses and between-group comparisons of *Δ* scores. Indeed, several studies have demonstrated that TR, particularly via the VRRS device, provides several advantages that may contribute to its effectiveness.^13,15,16^ Unlike traditional cognitive exercises, TR plus VR screen could create an immersive and interactive environment. This modality provides real-time feedback and personalization, which can improve patient engagement and cognitive stimulation.^30,31^ Previous studies have shown that interactive and engaging cognitive training can lead to more significant cognitive improvements than traditional methods.^13,30,31^ The ability of the VRRS to adapt exercises based on patient performance and provide immediate feedback could be a key role in the observed improvements in global cognitive functioning. In contrast, while traditional cognitive rehabilitation has also provided cognitive benefits, the lack of interactive and adaptive features may limit its effectiveness. Some studies have indicated that traditional paper-based exercises, while useful, may not offer the same level of engagement and cognitive challenge as interactive digital platforms.^12,13^ Therefore, the greater cognitive benefits observed in the EG underscore the potential benefits of incorporating advanced technologies into rehabilitation. As observed by previous studies, the use of multisensory feedback and the repeated performance of tasks with sensory stimulation contribute to promoting brain plasticity processes. VR activates mirror neurons and integrates perception, cognition, and action, thus improving the training effects and the patient's sense of self-efficacy.^32^

Another relevant aspect observed in our sample concerns the improvements in quality of life observed in the EG. The semi-immersive VR environment of the VRRS may facilitate greater patient engagement and motivation, leading to more extensive and intensive training.^33^ These aspects could have an important impact on quality of life due to increased awareness and control over their rehabilitation process.^34^ Previous research has shown that greater patient engagement and real-time feedback can significantly improve quality of life in various rehabilitation settings.^33,35^ TR plus VR can promote highly attractive, playful, and multidomain stimulation at the patient's home, avoiding the costs and logistical challenges associated with hospitalization or travel to our center as outpatients. This reduces patient stress and fatigue during training and potentially improves motivation and adherence to the treatment plan, with positive repercussions on patient well-being,^13^ as observed in our sample. Indeed, a lack of motivation in rehabilitation settings can often lead to poor adherence to prescribed exercise regimens and reduced training outcomes.^36^ Patients motivated by VR experiences may benefit from increased attention, which could lead to better functional outcomes and improved neurotransmission systems.^37^ This has clear benefits at the behavioral, and cognitive levels,^30,31,37^ as reported in the EG.

Finally, a reduction in caregiver burden was observed in both groups, but did not reach statistical significance. “Caregiver strain” refers to the multifaceted stress and challenges experienced by individuals providing care to someone with health needs. It thus encompasses the overall impact of caregiving on a person's well-being, often leading to negative outcomes such as burnout, depression, elevated stress levels, difficulty balancing care with other life responsibilities, and reduced quality of life.^38–41^ The observed reduction in caregiver strain could be attributed to the overall improvement in the patient's condition and the structured support provided through rehabilitation methods. This is in line with existing literature, which suggests that improvements in patient health and well-being often lead to a corresponding reduction in caregiver burden.^42,43^ Interventions that improve patient autonomy and reduce dependency have been shown to significantly alleviate the emotional and physical burden on caregivers.^44^ However, the limited sample size in our study may have limited the statistical power needed to detect significant changes, underscoring the need for further research with larger cohorts to conclusively determine these effects.

To sum up, the potential benefit of TR in improving both cognitive function and quality of life highlights its potential as a valuable tool in PD management. The VRRS's ability to provide customized, interactive, and engaging cognitive exercises could be a crucial factor contributing to these benefits. The real-time monitoring and feedback provided by the system also allow for personalized adjustments, potentially enhancing patient engagement and adherence.

This study has several limitations. First, the small sample size and short duration of the intervention may affect the generalizability of the results. Additionally, no formal sample size calculation was performed. As this was a pilot study, the sample size was determined based on feasibility and in line with existing methodological recommendations for preliminary trials. Nevertheless, the limited sample size reduces the statistical power of the analyses and warrants caution in interpreting the findings, which should be validated in future larger-scale studies. Moreover, the short duration of the intervention prevents us from assessing the sustainability of the observed improvements over time and thus limits our ability to draw conclusions about long-term efficacy. Future studies should include longer follow-up periods and a more heterogeneous patient sample in terms of disease stage, age, and cognitive reserve to improve generalizability. It is worth noting, however, that despite the limited sample size, participants in this study varied in disease severity and educational background, which may support the ecological validity and broader applicability of our findings. In any case, we noticed that given the small sample size, the study was adequately powered to detect only large effects (Cohen's *d* ≥ 1.36). Therefore, smaller but potentially clinically relevant differences may not have been identified. Another limitation is the use of a single cognitive screening tool (MoCA) rather than a comprehensive neuropsychological battery. While this choice was made to minimize patient burden in a pilot context, it may have limited the ability to detect specific domain-related effects of the intervention. Furthermore, although the usability of the intervention was evaluated using the SUS, only the total score was analyzed. This approach does not provide insights into specific dimensions such as learnability or comfort. Future studies should consider examining individual items or subscale trends to better capture the nuances of the user experience. Another limitation of this study is the difference in delivery mode between the two interventions. Although both groups received training targeting the same cognitive domains, the EG benefitted from interactive features such as real-time feedback and therapist-driven adaptation through the VR system. In contrast, the CG performed self-administered paper-and-pencil tasks without interactive components. This discrepancy may have contributed to the observed effects, making it difficult to isolate the specific impact of VR and tele-delivery alone. Finally, the lack of significant findings regarding caregiver burden suggests that further research is needed to explore the impact of TR on caregivers in more detail. Although no statistically significant effects emerged, a slight improvement was observed in the intervention group, suggesting a potential indirect benefit of cognitive TR on caregivers’ perceived strain. Future studies should consider adopting more sensitive or multidimensional tools to assess caregiver outcomes or integrate qualitative methods to better capture their lived experiences and subtle emotional changes. The study also did not assess long-term outcomes, which would be valuable in determining the sustainability of the observed benefits. Moreover, the absence of a mid-point or post-intervention follow-up limits our understanding of the trajectory and durability of these effects over time. Future studies should include multiple assessment points to better capture the persistence and clinical relevance of cognitive and quality of life improvements.

Future studies should consider larger sample sizes and longer intervention periods to validate and expand upon our findings. Additionally, research exploring the specific aspects of TR that most effectively reduce caregiver burden would be beneficial. Investigating the long-term effects of TR on both patients and caregivers will provide further insights into its potential as a sustainable intervention strategy for managing PD.

## Conclusion

In conclusion, TR is a feasible and a potentially alternative way to provide PD patients with cognitive rehabilitation at home. It could be an effective tool compared to traditional rehabilitation for PD patients, particularly in cognitive function and quality of life. While further research is needed, these findings could support the integration of TR into PD management strategies, highlighting its potential to improve patient outcomes and possibly reduce caregiver stress.

## References

[1] Jankovic J . Parkinson’s disease: clinical features and diagnosis. J Neurol Neurosurg Psychiatry 2008; 79: 368–376. 18344392 10.1136/jnnp.2007.131045

[2] Aarsland D Creese B Politis M , et al. Cognitive decline in Parkinson disease. Nat Rev Neurol 2017; 13: 217–231. 28257128 10.1038/nrneurol.2017.27 PMC5643027

[3] Baschi R Nicoletti A Restivo V , et al. Frequency and correlates of subjective memory complaints in Parkinson’s disease with and without mild cognitive impairment: data from the Parkinson’s disease cognitive impairment study. J Alzheimers Dis JAD 2018; 63: 1015–1024. 29710711 10.3233/JAD-171172

[4] Monastero R Cicero CE Baschi R , et al. Mild cognitive impairment in Parkinson’s disease: the Parkinson’s disease cognitive study (PACOS). J Neurol 2018; 265: 1050–1058. 29478221 10.1007/s00415-018-8800-4

[5] Martinez-Martin P Rodriguez-Blazquez C Kurtis MM , et al. NMSS Validation Group. The impact of non-motor symptoms on health-related quality of life of patients with Parkinson’s disease. Mov Disord Off J Mov Disord Soc 2011; 26: 399–406. 10.1002/mds.23462 21264941

[6] White DR Palmieri PA . There is “no cure for caregiving”: the experience of women caring for husbands living with Parkinson’s disease. Int J Qual Stud Health Well-Being 2024; 19: 2341989. 38657183 10.1080/17482631.2024.2341989 PMC11044767

[7] Nelson KE Runsabove K Saylor MA , et al. Predictors of supportive care needs during serious illness: cross-sectional analysis of reservation-based informal caregivers. J Hosp Palliat Nurs JHPN Off J Hosp Palliat Nurses Assoc 2024; 26: 273–281. 10.1097/NJH.0000000000001050 39106153

[8] Nasreen HE Tyrrell M Vikström S , et al. Caregiver burden, mental health, quality of life and self-efficacy of family caregivers of persons with dementia in Malaysia: baseline results of a psychoeducational intervention study. BMC Geriatr 2024; 24: 656. 39103767 10.1186/s12877-024-05221-9 PMC11301828

[9] Corallo F Maggio MG Bonanno L , et al. Burden in caregivers of patients with acquired brain injury: influence of family role and gender. NeuroRehabilitation 2024; 55: 69–76. 39031393 10.3233/NRE-240056

[10] Maggio MG Corallo F De Francesco M , et al. Understanding the family burden and caregiver role in stroke rehabilitation: insights from a retrospective study. Neurol Sci Off J Ital Neurol Soc Ital Soc Clin Neurophysiol 2024. 10.1007/s10072-024-07668-5 PMC11470910 38958795

[11] Maggio MG Baglio F Arcuri F , et al. Cognitive telerehabilitation: an expert consensus paper on current evidence and future perspective. Front Neurol 2024; 15: 1338873. 38426164 10.3389/fneur.2024.1338873 PMC10902044

[12] Maggio MG Luca A Cicero CE , et al. Effectiveness of telerehabilitation plus virtual reality (Tele-RV) in cognitive e social functioning: a randomized clinical study on Parkinson’s disease. Parkinsonism Relat Disord 2024; 119: 105970. 38142630 10.1016/j.parkreldis.2023.105970

[13] Maggio MG Cannavò A Quartarone A , et al. Enhancing the quality of life of patients with multiple sclerosis: promising results on the role of cognitive tele-rehabilitation plus virtual reality. Brain Sci 2023; 13: 1636. 38137084 10.3390/brainsci13121636 PMC10742306

[14] Olowoyo P Dhamija RK Owolabi MO . Telerehabilitation—historical perspectives and conceptual framework in reference to neurological disorders: a narrative review. NeuroRehabilitation 2025; 56: 5–18. 38995808 10.3233/NRE-240079 PMC11902888

[15] Bianchini E Onelli C Morabito C , et al. Feasibility, safety, and effectiveness of telerehabilitation in mild-to-moderate Parkinson’s disease. Front Neurol 2022; 13: 909197. 35785358 10.3389/fneur.2022.909197 PMC9245570

[16] Sanchez-Luengos I Balboa-Bandeira Y Lucas-Jiménez O , et al. Effectiveness of cognitive rehabilitation in Parkinson’s disease: a systematic review and meta-analysis. J Pers Med 2021; 11: 429. 34069980 10.3390/jpm11050429 PMC8157874

[17] Pazzaglia C Imbimbo I Tranchita E , et al. Comparison of virtual reality rehabilitation and conventional rehabilitation in Parkinson’s disease: a randomised controlled trial. Physiotherapy 2020; 106: 36–42. 32026844 10.1016/j.physio.2019.12.007

[18] Pareto L Johansson B Zeller S , et al. Virtual TeleRehab: a case study. Stud Health Technol Inform 2011; 169: 676–680. 21893833

[19] Postuma RB . Nonmotor aspects of Parkinson’s disease—how do they help diagnosis? Int Rev Neurobiol 2017; 133: 519–539. 28802931 10.1016/bs.irn.2017.04.002

[20] Lancaster GA Dodd S Williamson PR . Design and analysis of pilot studies: recommendations for good practice. J Eval Clin Pract 2004; 10: 307–312. 15189396 10.1111/j..2002.384.doc.x

[21] Nasreddine ZS Phillips NA Bédirian V , et al. The Montreal cognitive assessment, MoCA: a brief screening tool for mild cognitive impairment. J Am Geriatr Soc 2005; 53: 695–699. 15817019 10.1111/j.1532-5415.2005.53221.x

[22] Ferrario S Baiardi P Zotti A . Update on the family strain questionnaire: a tool for the general screening of caregiving-related problems. Qual Life Res Int J Qual Life Asp Treat Care Rehabil 2004; 13: 1425–1434. 10.1023/B:QURE.0000040795.78742.72 15503838

[23] Jenkinson C Fitzpatrick R Peto V , et al. The Parkinson’s disease questionnaire (PDQ-39): development and validation of a Parkinson’s disease summary index score. Age Ageing 1997; 26: 353–357. 9351479 10.1093/ageing/26.5.353

[24] Peto V Jenkinson C Fitzpatrick R . Determining minimally important differences for the PDQ-39 Parkinson’s Disease Questionnaire. Age Ageing 2001; 30: 299–302. 11509307 10.1093/ageing/30.4.299

[25] Brooke J . SUS—a quick and dirty usability scale. In: Jordan PW Thomas B Weerdmeester BA McClelland IL (eds) Usability evaluation in industry. UK: London Taylor & Francis, 1996, pp. 189–194. Available at: https://www.scirp.org/reference/referencespapers?referenceid=2552035

[26] Santangelo G Siciliano M Pedone R , et al. Normative data for the Montreal cognitive assessment in an Italian population sample. Neurol Sci Off J Ital Neurol Soc Ital Soc Clin Neurophysiol 2015; 36: 585–591. 10.1007/s10072-014-1995-y 25380622

[27] VRRS Evo [Internet]. Khymeia [cited 2025 July 23]. https://khymeia.com/it/products/vrrs-evo/

[28] Wong KP Tse M Qin J . Effectiveness of virtual reality-based interventions for managing chronic pain on pain reduction, anxiety, depression and mood: a systematic review. Healthcare 2022; 10: 2047. 36292493 10.3390/healthcare10102047 PMC9602273

[29] Chertkow H Nasreddine Z Joanette Y , et al. Mild cognitive impairment and cognitive impairment, no dementia: part A, concept and diagnosis. Alzheimers Dement J Alzheimers Assoc 2007; 3: 266–282. 10.1016/j.jalz.2007.07.013 19595948

[30] Marotta N Calafiore D Curci C , et al. Integrating virtual reality and exergaming in cognitive rehabilitation of patients with Parkinson disease: a systematic review of randomized controlled trials. Eur J Phys Rehabil Med 2022; 58: 818–826. 36169933 10.23736/S1973-9087.22.07643-2 PMC10081485

[31] Isernia S Di Tella S Pagliari C , et al. Effects of an innovative telerehabilitation intervention for people with Parkinson’s disease on quality of life, motor, and non-motor abilities. Front Neurol 2020; 11: 846. 32903506 10.3389/fneur.2020.00846 PMC7438538

[32] Born F Abramowski S Masuch M . Exergaming in VR: the impact of immersive embodiment on motivation, performance, and perceived exertion. In: 2019 11th International conference on virtual worlds and games for serious applications (VS-Games) [Internet], 2019 [cited 19 February 2025], pp. 1–8. Available at: https://ieeexplore.ieee.org/document/8864579

[33] Leonardi S Maggio MG Russo M , et al. Cognitive recovery in people with relapsing/remitting multiple sclerosis: a randomized clinical trial on virtual reality-based neurorehabilitation. Clin Neurol Neurosurg 2021; 208: 106828. 34332269 10.1016/j.clineuro.2021.106828

[34] Calabrò RS Naro A Russo M , et al. The role of virtual reality in improving motor performance as revealed by EEG: a randomized clinical trial. J Neuroengineering Rehabil 2017; 14: 53. 10.1186/s12984-017-0268-4 PMC5463350 28592282

[35] Brugada-Ramentol V Bozorgzadeh A Jalali H . Enhance VR: a multisensory approach to cognitive training and monitoring. Front Digit Health 2022; 4: 916052. 35721794 10.3389/fdgth.2022.916052 PMC9203823

[36] Hughes AJ Daniel SE Kilford L , et al. Accuracy of clinical diagnosis of idiopathic Parkinson’s disease: a clinico-pathological study of 100 cases. J Neurol Neurosurg Psychiatry 1992; 55: 181–184. 1564476 10.1136/jnnp.55.3.181 PMC1014720

[37] Touloudi E Hassandra M Stavrou VT , et al. Exploring the acute effects of immersive virtual reality biking on self-efficacy and attention of individuals in the treatment of substance use disorders: a feasibility study. Brain Sci 2024; 14: 724. 39061464 10.3390/brainsci14070724 PMC11274936

[38] Pigott JS Bloem BR Lorenzl S , et al. The care needs of patients with cognitive impairment in late-stage Parkinson’s disease. J Geriatr Psychiatry Neurol 2024; 37: 355–367. 38230692 10.1177/08919887231225484

[39] Pinquart M Sörensen S . Differences between caregivers and noncaregivers in psychological health and physical health: a meta-analysis. Psychol Aging 2003; 18: 250–267. 12825775 10.1037/0882-7974.18.2.250

[40] Rizzo A Sorrenti L Commendatore M , et al. Caregivers of children with autism Spectrum disorders: the role of guilt sensitivity and support. J Clin Med 2024; 13: 4249. 39064288 10.3390/jcm13144249 PMC11278243

[41] Mento C Rizzo A Settineri S . Caregivers help-seeking related to physical and mental burden. Clin Neuropsychiatry 2019; 16: 135–139. 34908948 PMC8650204

[42] Schulz R Sherwood PR . Physical and mental health effects of family caregiving. Am J Nurs 2008; 108: 23–27. quiz 27. 10.1097/01.NAJ.0000336406.45248.4c PMC2791523 18797217

[43] Gaugler JE Birkeland RW Albers EA , et al. Efficacy of the residential care transition module: a telehealth intervention for dementia family caregivers of relatives living in residential long-term care settings. Psychol Aging 2024; 39: 565–577. 38753405 10.1037/pag0000820 PMC11552057

[44] Gitlin LN Winter L Dennis MP , et al. A biobehavioral home-based intervention and the well-being of patients with dementia and their caregivers: the COPE randomized trial. JAMA 2010; 304: 983–991. 20810376 10.1001/jama.2010.1253 PMC4091681

